# News and Gossip

**Published:** 1870-08

**Authors:** 


### News and Gossip.

The distinguished surgeon, Prof. Syme, is dead.——Sir James Y. Simpson is to have a monument in Westminster Abbey.——Rokitanski has been elected President of the Imperial Academy of Sciences of Vienna.——Dr. J. L. Sullivan, of Mass., writes in defense of the American Medical Association, which is laudable, for *De mortuis nil*, etc.——One of our New York friends thinks one of the lamentable characteristics of American medical journalism is its "lack of independence—an almost childlike deference to traditional or self-constituted authorities—which, with few exceptions, is noticeable in our contemporaries." In a previous article, he thinks he would be content with the British system of medical education.——Dr. Paul Schœppe, of poisoning notoriety, has been refused a writ of error in Pennsylvania. His only chance now seems to be to "swear the peace" against the hangman.——Prof. Gross is not in favor of women as medical practitioners. President Stille favors petticoated medicine. Quite a wrangle was *gendered* in the Pennsylvania State Medical Society on this topic.——The *Nashville Medical Journal* comes to us in a new and neat dress, on which we congratulate it. Dr. Bowling is relieved from the cares and losses of publication, and thereanent, without the fear of the apostle before his eyes, he expresses himself facetiously. Drs. Nichol, Eve and Briggs have been added to the editorial force—Dr. W. Nichol being the managing editor. Whoever felicitates himself that Prof. Bowling has laid down his spear, we opine, however, would find himself mistaken, on occasion.——Dr. Peter, of Paris, prefers, in all cases, the Tinct. Iodinii, as a vesicant, to the cantharides.——It has been shown by M. Burral, before the Academy of Sciences at Paris, that rain water contains a notable quantity of phosphoric acid. By drinking 440 gallons of rain water, a man will imbibe from 13 to 15 grains of phosphorus; good news for the hydro- and homœopathists.——Large doses, internally, of crystallized carbolic acid are commended in variola at Paris. Better than this, the decrease of dyspepsia in that city is said to be attributable to the largely increased consumption of apples. One hundred millions of apples are digested in Paris every winter. This is probably the reason why it is written: "By their fruits ye shall know them," dyspepsia notably begetting bad temper.——Currents of electricity passed through the throat will often relieve severe hoarseness, at least temporarily. Hereafter, opera houses are to be furnished with a battery behind the scenes.——Dr. Lisle thinks arsenious acid will cure two-thirds of apparently hopeless cases of insanity. We think it will stop it in all cases, if the doses are large enough.——Dr. Thorne says permanganate of potash is *the* thing for oxaluria. He gives a teaspoonful three times a day of a solution containing four grains to the ounce of water.——Which reminds us that our friend Dr. Paoli thinks highly of this remedy in the treatment of rheumatism—so highly indeed, that he long since promised us an article on the subject.——Dr. Stivers, of San Francisco, eulogizes hydrate of chloral in full doses in delirium tremens, and Prof. Hammond, of New York, has favorable impressions from its employment in chorea. None of our correspondents as yet quote its employment in child-birth.——The *Indiana Med. Jour.* seems to regret that the Am. Med. Association failed to provide for compelling life insurance companies to pay their examiners the five dollar fees. Will the exponents of the Association tell us, "Why this is thus"?——Dr. Reynolds read a paper before the British Med. Association, highly recommending (in the usual terms, which see,) tincture of the perchloride of iron in rheumatic fever. No limitations as to the period of the disease. ——In the State of Michigan, clergymen and physicians live to 59 years, shoemakers 55½, and farmers 51 years. Dry goods clerks, however, measure off only 33.14 years.——Egyptian mummies are now regularly shipped to England for conversion into manure. The Pharaohs are said to be excellent fertilizers.——Dr. Tom O. Edwards, of Lancaster, Ohio, well known to the majority of our readers, reports in the *Bost. Jour. of the Gyn. Society*, a case of removal of a horse-shoe pessary from the bladder of a woman, where it had been introduced by a womb doctor, to relieve *prolapsus uteri.* This beats Prof. Chapman's case, where the student could not tell the same organs from a cart wheel. The womb doctor's explanation of the case was this: "I must have put one leg in the urethra, and as I reached for grease to anoint my fingers, the bladder must have sucked it in." Dr. T. O. E. fails to see the point of the explanation, but there must be one somewhere, as this makes four cases recorded of similar mal-introductions. We are obliged to add our own opinion, that in nine cases out of ten, when used, the horse-shoe or any other pessaries are as much out of place in the vagina, as in the bladder or œsophagus. In the great majority of cases which present themselves in private practice, we either advise, or, personally, dislodge the instruments, and toss them into the stove. — In the same *Journal* Dr. Jackson calls attention to the remarkable aphrodisiac powers of the *Helonias Dioica* or [false] unicorn root. He employs it in the dose of a dram of the powdered root three times a day. Dr. J. found this remedy capable of removing oxalate of lime from the urine in cases of that diathesis. Its use is suggested in loss of tone of the cremaster muscle, varicocele, relaxed vaginal parietes, etc. This article was first, permanently brought to the notice of the profession by Dr. Braman of Brighton (Bost. M. & S. Journal, v. XL., p. 416) in 1849. Prof. Charles A. Lee subsequently eulogized it—the eclectics rejoiced in it, and now the *Chic. Med. Jour.* pats its head and dismisses it for its travels, through the press and people, for its nine days' new lease of life.——"The *country members*," a medical review of that excellent little book, *A Physician's Problem*, thinks, should have had the benefit of translations of the frequent quotations in Latin, French and other languages. We will wager a copy of Wood's Pocket Medical Lexicon, that that reviewer has never lived in the country, and knows nothing of the class of men he impliedly sneers at. Let us inform him that in this country the rural practitioners, as a body, surpass their city brethren in both general and professional knowledge. In the country, and particularly in small villages of a few thousand inhabitants, a man's real qualifications are scrutinized with a closeness and sharpness impossible in the city. If a doctor wishes to conceal his ignorance, let him go to the larger cities where *deportment* will answer his turn with the flimsiest possible acquaintance with his business. But unless thoroughly *posted*, let him beware of the town of from 2,500 to 10,000 inhabitants. He must run the gauntlet of the Committee of the Whole, ready to resolve themselves into a Committee of Safety. Men will not be wanting, apparently plain men, who will see "through and through" him. In the great city, nobody has time for his analysis, unless he foolishly invokes it by presumption.

### Medical Witnesses.

A medical witness would do well to remember, 1st, That no one has a *right*, as the law now stands, to ask an *opinion* of him; 2d, That, if asked for an opinion, he may refuse to give it; and 3d, That, in order to throw doubt on his testimony, the opposing counsel will, at times, endeavor to entrap him into the expression of an opinion, and afterwards, by subsequent questions, to lead him to absurd conclusions, whereby his testimony, although strictly accurate, may be made to appear wrong in the eyes of the jury. 4th. In the statement of facts, it would be well to be as brief as is consistent with clearness. Moreover, the witness should, as far as possible, avoid all technical language. For example, a witness, when about to instruct a jury, will lose credit for clearness of statement, and may darken the mind of the whole court, by telling of the *scapula* instead of the shoulder-blade, or using *clavicle* for collar-bone, *sternum* for breast-bone, etc. 5th. If he be not sure that he is really an expert in the matter in hand, he should beware of going upon the stand at all. If he do so, he deserves, and probably will get, a severe castigation from opposite counsel, and he has no right to ask sympathy from his professional associates. He cannot blame lawyers for even apparent rudeness in their attempts to prove him, before the jury, to be the ignoramus he really is. 6th. Some, while on the stand, seem to forget the great difference there is between the statement of a fact, or of a series of facts, and the inferences that may be made from these facts. The facts should be given, the inferences may be left to the wisdom of the court or jury to make. If the witness makes them, he does so at the risk of severe criticism.—*Boston Med. Jour.*

---

### First Meeting of the North-Eastern Indiana Medical Soc.

Pursuant to a call for the purpose of organizing a Medical Society to embrace the counties of LaGrange, Steuben, Noble and DeKalb, the following named physicians met on Wednesday, June 15th, at the parlor of the Tremont House, Kendallville, Ind.:

Drs. Denny, Carr, Crum, Palmiter, Randall, Landon, Ligonier; Franks, Adair, Brimfield; Fansler, Rome City; Ward, Wawaka; Denny, Hawpatch; Wood, Angola; Dancer, South Milford; Wright, Avilla; Vincent, Abell, Williams, Gilbert, Kendallville.

After reading the minutes of the preliminary meeting held May 20th, 1870, the committee on permanent organization, consisting of Drs. Denny, Franks, and Palmiter, reported the following officers, who were promptly elected:

President—O. J. Vincent. Vice-Presidents—Wood, Dancer, Stough, Palmiter. Secretary—J. L. Gilbert. Corresponding Secretary—D. W. Crum. Treasurer, L. F. Abell. Censors—Drs. Carr, Williams, Franks, Denny and Landon.

On motion, the Chair appointed Drs. Denny, Wood and Crum to prepare a Constitution and By-Laws.

They reported immediately, and a Constitution and By-Laws were adopted, of which the Secretary was ordered to have one hundred copies printed. A fee-bill was then adopted, and two hundred copies ordered to be printed in card form.

The following resolutions were adopted and ordered to be published in the papers of the counties of Steuben, LaGrange, DeKalb and Noble:

*Resolved*, That all who dishonestly or maliciously refuse to pay their doctor bills be reported to this society.

*Resolved*, That no member of this society shall furnish medical aid, except in extreme cases, to persons thus reported, until they have paid their former doctor bills, unless they pay a double fee in advance.

*Resolved*, That the honest poor are always the proper subjects for our sympathy.

The code of ethics of the American Medical Association was adopted. On motion, Drs. Landon and Wood were requested to prepare essays to be read at the next meeting.

Scarlatina and Diphtheria were selected as subjects for discussion at the next meeting.

On motion, the Secretary was ordered to publish the proceedings of this meeting in the journals of the four counties named above, and in the medical journals of Chicago and Cincinnati.

The society then adjourned, to meet at Waterloo, on the first Tuesday of September next. O. J. VINCENT, *Pres.*

J. L. GILBERT, *Sec.*

### *American Pharmaceutical Association. Notice of Annual Meeting.*

The 18th Annual Meeting of the American Pharmaceutical Association will be held in the city of Baltimore, on the second Tuesday (the 13th day) of September, 1870, commencing at 3 o'clock, P. M. The place of meeting and the arrangements for the accommodation of those who attend, will be announced by the local Secretary. It is expected that the several standing and special committees will be prepared with full and highly instructive reports on the many important subjects which should engage our earnest attention at this time.

The central position of Baltimore will afford opportunity for many to be present at this meeting who have not usually met with us. A full attendance of the members is earnestly desired, as, among the important business to be brought forward, will be the revision of the constitution more particularly in reference to improving the financial status of the Association.

Pharmacists and druggists eligible to membership are earnestly invited to forward or to present their names for election and thereby aid in extending the usefulness of this Association. Those desiring to join can obtain the necessary blank applications from the chairman of the Executive Committee, Thomas S. Wiegaud, 528 Arch street, or from the permanent Secretary, John M. Maisch, 1607 Ridge avenue, Philadelphia. A cordial invitation is extended to all engaged in manufactures in any way connected with Pharmacy or Chemistry, to send specimens of their productions for exhibition during the session. These may be sent to Prof. J. Faris Moore, local Secretary, Baltimore, accompanied with an invoice and a full description of the articles sent. E. H. Sargent, *President.*

Chicago, July 1, 1870.

### *Obituary.*

J. S. Hildreth, M.D., formerly Superintendent of the Dessuarres Eye and Ear Hospital, occupying the Cook County Hospital building in this city during the war, and recently engaged in the practice of that specialty at his residence, 526 Wabash avenue, died suddenly July 22. It is understood that he had been suffering severely from neuralgia, and that his death was due to an overdose, accidentally taken, of some powerful narcotic. Dr. H. was the son-in-law of the Hon. Jacob M. Howard, of Detroit, one of the U. S. senators from Michigan. His associates on the medical staff of Cook County Hospital adopted the following resolutions:

*Whereas,* We have just learned of the sudden death of our friend and associate, J. S. Hildreth, M.D.; therefore

*Resolved,* That in the death of Dr. Hildreth the medical board has lost one of its most worthy and industrious members, the profession of our city one of its brightest ornaments, and society a gentleman of high culture and excellent social qualifications.

*Resolved,* That in common with the entire profession, we tender to his afflicted family the assurance of our high respect for the deceased, and our heartfelt sympathy for them in this their irreparable loss.

*Resolved,* That the board attend the funeral in a body, and ask permission to render upon that occasion such services as the family may designate.

*Resolved,* That a copy of these resolutions be sent to each of the daily papers of this city, and to the medical journals for publication; a copy transmitted to the family of the deceased, and also that a copy be preserved in the archives of this hospital.

